# Current Paradigm and Future Directions in the Management of Nodal Disease in Locally Advanced Cervical Cancer

**DOI:** 10.3390/cancers17020202

**Published:** 2025-01-09

**Authors:** Elki Sze-Nga Cheung, Philip Yuguang Wu

**Affiliations:** Department of Clinical Oncology, Pamela Youde Nethersole Eastern Hospital, Hong Kong, China; csn891@ha.org.hk

**Keywords:** cervical cancer, radiotherapy, simultaneous integrated boost, intensity-modulated radiotherapy, nodal control, immunotherapy

## Abstract

Approximately 36% of patients with cervical cancer present with regional nodal metastasis at diagnosis, which is associated with adverse survival outcomes after definitive treatment. In the modern era of chemoradiotherapy (CRT) and image-guided adaptive brachytherapy (IGABT), where excellent local control is achieved for patients with locally advanced cervical cancer (LACC), nodal failure remains a major challenge to cure. To optimize treatment outcomes for node-positive LACC and reduce the incidence of nodal failure, various treatment approaches have been explored, including methods of surgical nodal staging or dissection, RT dose escalation strategies, such as intensity-modulated radiotherapy (IMRT) with simultaneous integrated boost (SIB) to involved nodes, and elective treatment of subclinical para-aortic (PAO) disease. Additionally, there is growing interest in emerging precision RT techniques, such as magnetic resonance-guided radiotherapy (MRgRT) and proton therapy, which may allow for further improvement in the therapeutic ratio. This review outlines the various methods of detection of nodal metastasis, treatment options for node-positive LACC, techniques of nodal radiotherapy and their clinical evidence in efficacy and toxicity profiles. Furthermore, recent advances in systemic therapy and promising novel therapeutic directions that may shape the management of node-positive LACC are discussed.

## Simple Summary

Approximately 36% of patients with cervical cancer present with regional nodal metastasis at diagnosis, which is associated with adverse outcomes after definitive chemoradiotherapy. Various approaches to treatment optimization have been explored, including surgical nodal staging or dissection, radiotherapy dose escalation strategies, such as intensity-modulated radiotherapy with simultaneous integrated boost to involved nodes, and elective treatment of subclinical paraaortic disease. This review provides an overview of the current paradigm in the management of node-positive cervical cancer, including nodal staging, node-directed treatment approaches, as well as recent advances in systemic therapy. Moreover, future directions in personalized therapy to maximize the therapeutic ratio of radiotherapy and tailor treatment with predictive biomarkers are discussed.

## 1. Introduction

Locally advanced cervical cancer (LACC) represents a substantial proportion of all cervical cancer, estimated at 37% globally, with the highest proportions (≥50%) reported in Asia, Europe, Brazil, and Morocco [1]. In the past decade, there has been a paradigm shift in the standard of care from three-dimensional conformal radiation therapy to intensity-modulated radiation therapy (IMRT) for external beam radiotherapy (EBRT) and from a historical two-dimensional point-based approach to modern three-dimensional image-guided adaptive brachytherapy (IGABT). Notably, the employment of IGABT has benchmarked excellent three-year local and pelvic control rates within the ranges of 91–95% and 87–97%, respectively [2,3,4]. In this modern era of IGABT with well-controlled primary tumors [2,5], regional nodal and distant metastasis have become the predominant forms of treatment failure and constitute major challenges to cure [6]. It is particularly challenging in the subgroup of LACC with nodal involvement, which accounts for approximately 36% of cervical cancer patients, as they are known to have increased risks of all failure types [6,7], underscoring the critical need for the further optimization of treatment strategies.

Approaches to optimize the management of nodal disease in LACC include surgical nodal staging or dissection, escalation of nodal irradiation during EBRT, and enhancement of systemic treatment. Dose escalation to macroscopic nodes [8,9,10,11,12,13,14,15,16] and elective extended field para-aortic (PAO) irradiation [8,10,12,13,15] to eradicate subclinical disease have been investigated and are increasingly practiced with contemporary radiotherapy techniques, which allow conformal dose prescription with a minimal increase in toxicity. Furthermore, recently published pivotal studies, such as INTERLACE [17] and KN-A18 [18], have transformed the use of systemic therapy for LACC in the context of definitive radiotherapy.

Moving forward, emerging novel precision radiotherapy techniques such as magnetic resonance-guided radiotherapy (MRgRT) and particle therapy present opportunities to enhance the therapeutic ratio in radiotherapy. In addition, ongoing research efforts to delineate the possible synergy between radiotherapy and immunotherapy hold great potential to redefine radiotherapy and systemic therapy combinations. With the rapid emergence of artificial intelligence, deep learning applications, and realization of molecular biomarkers, there is promise for the development of a personalized approach in the management of node-positive LACC.

This review aims to comprehensively discuss the current landscape of nodal management in LACC treated with definitive chemoradiotherapy (CRT), especially in the context of contemporary image-guided IMRT and IGABT, as well as the challenges and future directions in this evolving field.

## 2. Incidence and Distribution Pattern of Nodal Metastasis

The incidence of pelvic and/or paraaortic (PAO) nodal metastases in cervical cancer at the time of diagnosis varies with primary tumor extent [7,19] and is influenced by the staging modality utilized. Accurate estimation of real-world incidence of nodal disease is limited by the lack of nodal information in the previous International Federation of Gynecology and Obstetrics (FIGO) staging system [20], resulting in inconsistent reporting in clinical practice, as well as limited use of advanced imaging in the past.

In a recent Danish prospective multicenter study [19] on women with early T-stage cervical cancer receiving radical surgery and sentinel lymph node mapping, the incidence of pelvic lymph node metastasis detected from staging surgery was 6.0% for FIGO-2018 stage IB1 disease, 24.5% for stage IB2, and 14.3% for stage IB3, respectively. Risk factors significantly associated with nodal metastasis were FIGO-2018 stage ≥IB2, a proportionate depth of tumor invasion >2/3, and lymphovascular space invasion. In patients with LACC receiving definitive CRT, a similar correlation in the incidence of regional nodal involvement by local tumor stage is seen. In the EMBRACE I study [7], rates of nodal involvement at diagnosis across different FIGO-2009 stages were 36% for IIA, 45% for IIB, 58% for IIIA, 61% for IIIB, and 62% for IVA disease.

In cervical cancer, nodal spread typically follows an orderly pattern from the lower pelvic region (including parametrial, internal iliac, external iliac, obturator, or presacral nodes) to common iliac and PAO regions [21]. PAO involvement is usually accompanied by positive pelvic nodes, with ‘skip metastasis’ occurring typically below 4%, as reported by previous surgical series [22,23]. The EMBRACE I study [7] revealed that nearly all (99%) node-positive patients exhibited affected lymph nodes in the pelvis, with common iliac (30%) and external or internal iliac (92%) being common sites. PAO and inguinal lymph nodal involvement were observed in 14% and 3% of node-positive patients, respectively. Notably, 37% of node-positive patients had nodes involved in multiple nodal regions. Any nodal spread beyond the defined regional nodal sites is considered distant nodal disease. Despite being categorized as distant metastasis, nodal involvement in the inguinal region can still be treated with a curative intent [7,8,14,24].

## 3. Staging and Detection of Nodal Disease in LACC

### 3.1. Prognostic Impact of Nodal Metastasis in LACC

A major update in the FIGO-2018 staging system [25] was the introduction of stage IIIC, which encompasses patients with pelvic (IIIC1) and/or PAO lymph node (IIIC2) metastasis, irrespective of primary tumor size or extent. Radiological findings from various imaging modalities, such as ultrasound, computed tomography (CT), magnetic resonance imaging (MRI), positron emission tomography-computed tomography (PET-CT), or PET-MRI, can be used to assign stage.

Patients with node-positive LACC face increased risks of all types of failure [6,7,26] and poorer survival [25,27,28,29]. Analysis from the RetroEMBRACE study [6] revealed that patients without and with pelvic lymph node metastasis had incidences of local, regional, PAO, and distant failures of 8.3% vs. 11.5%, 3.0% vs. 8.2%, 6.4% vs. 10.2%, and 14.7% vs. 27%, respectively, after a median of 43 months following definitive CRT and IGABT. Within the EMBRACE I prospective study [7], with 1338 patients analyzed, the 5-year nodal control was 93% in the node-negative group and 81% in the node-positive group, i.e., an absolute difference of 12%. The 5-year overall survival (OS) was 82% for node-negative and 68% for node-positive patients. In particular, the presence of PAO involvement indicates worse prognosis compared to pelvic lymph node-only disease [7,30].

It is crucial to note that FIGO IIIC cervical cancer patients exhibit significant heterogeneity in clinical outcomes that are known to be strongly influenced by the extent of the local tumor [31] and, albeit less documented, nodal burden [32,33,34]. In various validation studies, higher FIGO-2018 stages were not consistently correlated to worse survival [30,31,35]. Wright et al. [30] reported 5-year survival rates of 40.7% for stage IIIA, 41.4% for IIIB, 60.8% for IIIC1, and 37.5% for IIIC2. Stage IIIC1 showed superior survival rates compared to IIIA and IIIB, while IIIC2 had survival rates overlapping with IIIA and IIIB, highlighting the additional prognostic significance of local tumor extent in stage III patients. Despite the inclusion of node-positive patients into one FIGO-2018 stage category, both local tumor and nodal characteristics should be considered to provide adequate prognostic precision to be clinically meaningful.

### 3.2. Imaging in Nodal Staging

Conventional imaging (MRI or CT) evaluates lymph nodes based on their size and morphological criteria. Traditionally, size has been the primary criterion for diagnosing nodal involvement in cervical cancer, where lymph nodes larger than 10 mm in short axis or greater than 8 mm in short axis with a rounded morphology are considered worrisome [36]. In a meta-analysis evaluating the nodal staging performance, CT and MRI show high pooled specificity (92–95%) but relatively poor sensitivity (50–56%), as normal-sized nodes can also harbor metastatic disease [37]. It has also been shown that the addition of diffusion-weighted imaging (DWI) to T2-weighted MRI results in a higher pooled sensitivity of 84% versus 50% in conventional MRI [38].

18F-fluorodeoxyglucose (FDG) PET-CT has an established role in disease staging and therapy planning in patients with LACC, particularly in the depiction of nodal disease and distant metastases [39]. It is able to detect metastatic disease even in normal-sized nodes by demonstrating high tumor-related metabolism. This results in an improved nodal detection sensitivity of 80–91% [40,41,42]. The use of FDG PET-CT for staging in LACC has been shown to prompt a change in treatment or radiotherapy plan in 18–45% of patients [43,44]. The ESGO-ESTRO-ESP cervical cancer guidelines thus recommend whole-body PET-CT staging in LACC before definitive CRT [45].

### 3.3. Controversies Regarding Surgical Nodal Staging

There have been years of debate regarding the role of surgical staging before definitive radiotherapy in LACC. Central to this discussion is the concern pertaining to the limitations of imaging modalities to accurately detect nodal disease and that occult nodal metastasis falling below the detection threshold of imaging may result in under-treatment and poor outcomes. This is particularly relevant in the PAO region, which is beyond the standard pelvic radiotherapy portal. Even with PET-CT, the false negative rate for the detection of PAO involvement ranges from 8–12% overall, increasing to 22–24% in patients with pelvic nodal disease [23,46]. Some would therefore advocate pre-therapeutic surgical PAO staging to delineate the extent of nodal disease and customize radiotherapy plans with the dual objective of extending the radiation field in patients with microscopically positive PAO disease while sparing patients from the additional morbidity of PAO irradiation in those negative for involvement. In an earlier retrospective pooled analysis of 685 patients that received CRT in Phase III GOG trials (GOG 85, GOG 120, and GOG 165) [47], the outcomes of patients who had negative PAO status determined by surgical sampling were compared to those who had radiographic (CT or MRI) staging alone. Pre-radiotherapy surgical exclusion of PAO disease was associated with better prognosis and lower rates of PAO relapse compared to radiographic exclusion. In multivariable analysis, radiographic staging alone was associated with worse progression-free survival (PFS) (HR 1.35, 95% CI: 1.01–1.81) and OS (HR 1.46, 95% CI: 1.08–1.99). On the contrary, in a randomized trial involving surgical versus clinical staging in 61 patients with LACC, surgical nodal staging was associated with significantly worse PFS and OS, and the trial was terminated early based on futility [48]. Another prospective study conducted in three French comprehensive cancer centers evaluated the impact of pre-therapeutic laparoscopic PAO staging in LACC with negative PAO involvement determined by PET-CT [49]. Radiation fields were extended to the PAO region when PAO nodes were identified. In the total of 237 patients included, 12% were found to have nodal involvement at staging surgery, representing false-negative PET-CT. Event-free survival was found to be similar in patients without PAO disease compared to those with PAO metastasis ≤5 mm (3-year event-free survival of 74% and 69%, respectively), suggesting a potential role of surgical staging in identifying occult PAO metastasis that could be effectively treated with subsequent extended-field CRT. However, it is noteworthy that this was a single-arm study for which all patients underwent surgical nodal staging, and outcomes of patients who did not undergo surgery were not reported, nor did the study address the role of elective PAO irradiation.

The most compelling evidence to date on the role of surgical nodal staging in LACC was from the multi-institutional randomized controlled trial, Uterus 11 [50], which enrolled 255 patients with FIGO-2009 stage IIB-IVA cervical cancer. Patients were randomized to either radiological staging alone or laparoscopic transperitoneal surgical staging, which entailed complete bilateral pelvic and PAO lymphadenectomy up to the level of the renal vein. Subsequently, all patients underwent definitive CRT and brachytherapy. Notably, extended-field radiation therapy was only administered to patients with positive PAO lymph nodes, whether pathologically or radiologically confirmed, while elective PAO irradiation was not permitted. The overall incidence of any postoperative complication was low (7.3%). There was also no difference in the time to definitive CRT initiation between the two groups (median: 13 vs. 13.5 days from randomization in the surgical and radiological groups, respectively), supporting that laparoscopic PAO staging in experienced centers is not associated with significant morbidity or impacts the patient’s ability to receive subsequent CRT. On the other hand, although the surgical staging arm exhibited a significant upstaging rate (33%) compared to radiological staging alone, there was no difference in the disease-free survival (DFS) or OS between the two groups after a median follow-up of 90 months. Furthermore, the high rates of distant metastasis in both groups challenge the value of surgical PAO staging in terms of overall treatment outcomes. Caveats of this study included significant underutilization of staging PET-CT and a lack of patient-reported outcomes.

Historically, surgical nodal staging was performed via transperitoneal laparotomy [51], which was later replaced by laparoscopic techniques [23,52,53,54]. The transperitoneal approach provides adequate working space with recognizable anatomical landmarks but may necessitate bowel mobilization. In contrast, the extraperitoneal approach reduces the risk of bowel injury and adhesion; thus, it is feasible even after previous abdominal surgery, although it is limited by a confined working space with a higher risk of disorientation [52]. A recent systematic review and meta-analysis [53] demonstrated that the extraperitoneal approach is safe and efficient, with lymph node yield comparable to the transperitoneal approach and with shorter operative times and lower intraoperative complications. Concerns with surgical nodal staging include surgical morbidity, which varies with surgical approach (open [51] or laparoscopic [23,54,55,56], transperitoneal [54] or extraperitoneal [57,58]), and consist of wound complications, ureteral and vascular injury, and lymphocysts, with overall incidence reported between 7–19% [23,51,54,55,56,59]. Furthermore, the potential delay in definitive CRT, leading to prolonged overall treatment times (OTT), variable surgical quality, the lengthy professional learning curve for minimally invasive PAO lymphadenectomy, and the lack of survival benefit demonstrated from clinical trials have all limited the widespread application of surgical PAO staging in clinical practice.

The current ESGO/ESTRO/ESP guidelines on cervical cancer [45] include the option of PAO nodal dissection to assess the need for elective PAO radiotherapy in patients with negative PAO nodes and positive pelvic nodes on imaging, as a grade C recommendation. While the debate surrounding surgical PAO nodal staging continues, there is a growing utilization of elective PAO irradiation in patients with pelvic nodal disease and high risk of occult PAO metastasis based on clinical factors. Rapid advancements in radiotherapy techniques have allowed the conformal delivery of PAO irradiation without significant increase in treatment morbidity [60,61,62]. Ongoing prospective clinical trials, including PAROLA [63] and NCT05378087 [64], will further address the value of surgical staging compared to radiological staging for pelvic node-positive LACC in the context of PET-CT and elective PAO radiotherapy.

## 4. Node-Directed Treatment Intensification Strategies in LACC

Within the EMBRACE I study, 5-year nodal control was 93% in node-negative and 81% in node-positive patients, reflecting a substantial 12% absolute difference [7]. This highlights the importance of node-directed treatment intensification to improve outcomes. Without consensus on the optimal management, various approaches have been explored, including surgical debulking prior to definitive CRT, radiotherapy dose escalation in the form of sequential boost (SEB) or simultaneous integrated boost (SIB), or a combination, with clinical practices heterogeneous across institutions. Relevant clinical evidence to support these approaches is summarized in this section.

### 4.1. Surgical Debulking of Macroscopic Nodes Prior to Definitive CRT

The rationale for the surgical debulking of macroscopically enlarged lymph nodes detected by clinical assessment prior to definitive CRT is based on nodal failure patterns in node-positive LACC, and that conventional EBRT doses (45–50.4 Gy) may produce insufficient tumoricidal effect, particularly in the presence of bulky nodes. Gross macroscopic debulking of enlarged nodes may increase chances of sterilization by CRT and reduce the potential toxicity from higher radiation doses. Early experience from the 1970s to 1990s [65,66] illustrated the feasibility of debulking of bulky lymph nodes detected on radiological assessment or at the time of radical hysterectomy, prior to EBRT, with overall low rates of surgical morbidity. The survival of patients with completely resected bulky pelvic and common iliac nodes was found to be similar to that of patients with microscopic nodal disease, implying a potential therapeutic effect of debulking surgery. A retrospective mono-institutional study reported on the outcomes of 266 patients with FIGO IB-IV cervix cancer treated between 1978 and 1990 who received extraperitoneal pelvic and PAO lymphadenectomy for staging or debulking purposes prior to definitive radiotherapy [66]. Patients with node-positive disease received extended field PAO irradiation. In this study, 5- and 10-year DFS were found to be similar in the patients with microscopic or macroscopically resected nodal disease (43% and 35% vs. 50% and 46%, respectively), while all patients with unresectable nodal disease recurred within 3 years following radiotherapy. The incidence of RTOG grade 4 treatment-related morbidity was 10.5%, mainly attributed to bowel complications. Although an overall low incidence of treatment-related mortality was recorded (1.1%), two patients who had resected macroscopic nodes followed by extended field PAO irradiation died due to bowel perforation. Other complications included symptomatic lymphocysts requiring drainage or laparotomy in 6.7% of patients and lymphedema occurring in 18.4%. It is noteworthy that these retrospective cohorts included patients who received treatment in an era of low availability of diagnostic imaging, such as CT or MRI, traditional radiotherapy techniques, and concurrent chemotherapy, which were yet to become the standard of care in LACC. Nevertheless, the encouraging tumor control outcomes, including long-term survival observed in a subset of patients with resected bulky or matted pelvic or PAO nodes, reflect a potential role of pretherapeutic nodal debulking to reduce tumor burden before definitive CRT, albeit at the expense of increased treatment-related morbidity.

More recent data from high-volume centers suggest that the resection of bulky pelvic nodes can be performed via the laparoscopic approach without a significant increase in surgical morbidity or disruption to the initiation of radiotherapy [67,68]. Retrospective analysis of patients who received debulking of enlarged pelvic lymph nodes during pretherapeutic laparoscopic PAO nodal staging from 11 specialized gynecologic oncology units in Spain showed an overall low incidence of surgical morbidity in patients who had additional nodal debulking, and no difference was observed compared to patients who only had PAO staging surgery (incidence of intraoperative complications 3.7% vs. 2.7%, *p* = 0.744; early postoperative complications 8.0% vs. 6.3%, *p* = 0.776; late postoperative complications 6.1% vs. 2.7%, *p* = 0.252) [57]. However, no definite survival advantage of this approach was demonstrated. Based mostly on retrospective evidence, the practice of therapeutic nodal debulking surgery has been heterogeneous [7,69,70] and mostly limited to centers with surgical expertise. Recent reports have suggested that surgical debulking with varied extent and techniques, EBRT boost, or a combination of such may be practiced in real-world settings. Analysis of institutional data from Vienna pertaining to patients included in RetroEMBRACE and prospective EMBRACE I and II studies demonstrated that regional nodal control was improved in patients who received lymph node surgery or radiotherapy boost in addition to standard CRT [70]. A recent retrospective analysis of 190 patients with node-positive LACC from The Netherlands Cancer Registry compared survival and toxicity outcomes between surgical debulking, radiotherapy boost, or no additional node-directed therapy [71]. From this study, no advantage in relapse-free or overall survival was observed from either node-directed intensification strategy, while radiotherapy boost was associated with a lower incidence of treatment-related toxicity compared to debulking surgery. Drawbacks of the study were a lack of details on radiotherapy techniques and boost dose prescription, limiting the interpretation of the relative efficacy of the boost strategy. Without high-quality prospective evidence, the surgical removal of large pathological pelvic and/or PAO nodes before definitive CRT is not routinely recommended by the ESGO/ESTRO/ESP guidelines. A multicenter randomized controlled trial (CQGOG0103) [72] in China plans to compare the role of pelvic and PAO lymph node dissection before CRT to definitive CRT with nodal boost (55–60 Gy) in patients with FIGO IIICr LACC and a short diameter of ≥15 mm based on imaging. The results from this study will hopefully shed light on the optimal treatment strategy in this controversial area.

### 4.2. External Beam Radiotherapy Boost to Macroscopic Nodes

In recent years, nodal treatment intensification by EBRT dose escalation has been a subject of growing interest. Although a clear dose-response relationship for nodal control has not been defined, resulting in a lack of consensus on the optimal dose prescription, dose escalation to clinically involved nodes from 45–50 Gy to 55–60 Gy in EQD2 (α/β = 10) has been shown to improve regional nodal control [14] and DFS [73] without significant additional toxicity across retrospective and prospective studies and is commonly delivered in clinical practice. Bacorro et al. [74] found that increasing the total nodal dose from 50 to 60 Gy in EQD2 could significantly improve nodal control, especially for bulky nodes. However, doses exceeding 60 Gy would raise concerns of increasing morbidity, especially when applied in the PAO region, and data on its safety are scarce [75]. From retrospective series, it was also suggested that the dose–response relationship of involved nodes appears to be flat at 55–60 Gy; thus, the benefit of further dose escalation beyond 60 Gy is doubtful [9]. Depending on the size and location of individual nodes and the brachytherapy technique, it is estimated that dose contribution from brachytherapy to lymph nodes in the external and internal iliac regions is around 5 Gy for ilio-obturator lymph nodes, 2.5 Gy for common iliac lymph nodes, and negligible at PAO nodes, and this dose should be accounted in nodal dose prescriptions [76,77,78].

Analysis of patients in the EMBRACE I prospective study showed the crude nodal failure rate was 16% for patients with node-positive disease [7]. Nodal boost (a mean total dose of 59 Gy) was delivered to 66% of node-positive patients, resulting in a crude nodal failure rate of 15% compared to 17% for node-positive patients treated without a boost [7]. Of the node-positive patients treated with nodal boost, approximately 12% developed failures within the boost target volume, suggesting a potential benefit from nodal dose escalation [7]. However, it is worthwhile to note that the nodal disease outcome was not the primary endpoint of EMBRACE I, and analysis of nodal failure based on individual nodal characteristics or dose-volume parameters was not feasible. Furthermore, clinical practices varied significantly between study centers, allowing a wide range of techniques of SEB or SIB, dose prescriptions ranging from 50–67 Gy, and the combination treatment of surgery with nodal boost in many patients.

From available evidence, factors adversely influencing the control of involved nodes include nodal size (e.g., >2 cm [79,80]) or volume (e.g., >3 cm^3^ [14]), a prescription dose of less than 57.5 Gy in EQD2 [74], and poor early nodal regression after EBRT [81]. Furthermore, a significant correlation has been observed between high SUVmax and treatment failure in boosted nodes [24], suggesting that intensifying treatment for nodes with high PET avidity may be beneficial.

Early data on nodal boosting mainly involved SEB [61,73], with the ability to deliver an additional dose adapted to the nodal response after EBRT. With evolving radiotherapy techniques and the adoption of IMRT or volumetric modulated arc therapy (VMAT) as the standard of care in many parts of the world, SIB has been increasingly utilized to deliver dose escalation [8,10,11,12,13,14,16,82]. The most prominent advantage of SIB is that it offers potential advantages of a higher dose per fraction, resulting in higher biologically equivalent doses (BED) without increasing OTT, which limits tumor repopulation, and the boosted nodes can also benefit from concurrent chemotherapy. Dosimetric studies have also reported high dose conformality, superior organ-at-risk (OAR) sparing, and better control of hotspots compared to SEB [83].

**Figure 1:**
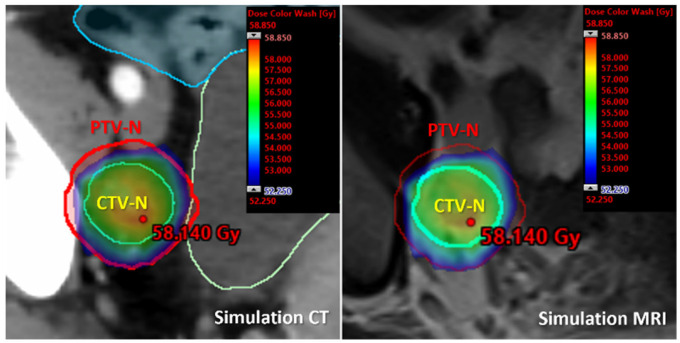
Coverage-probability-based treatment plan using VMAT for simultaneous integrated boost of a pathological right external iliac node. PTV-N is 5 mm margin from CTV-N. Boost dose of 55 Gy is prescribed with the elective dose of 45 Gy in 25 fractions. Dose color wash represents 95–107% of the prescribed dose.

**Figure 2:**
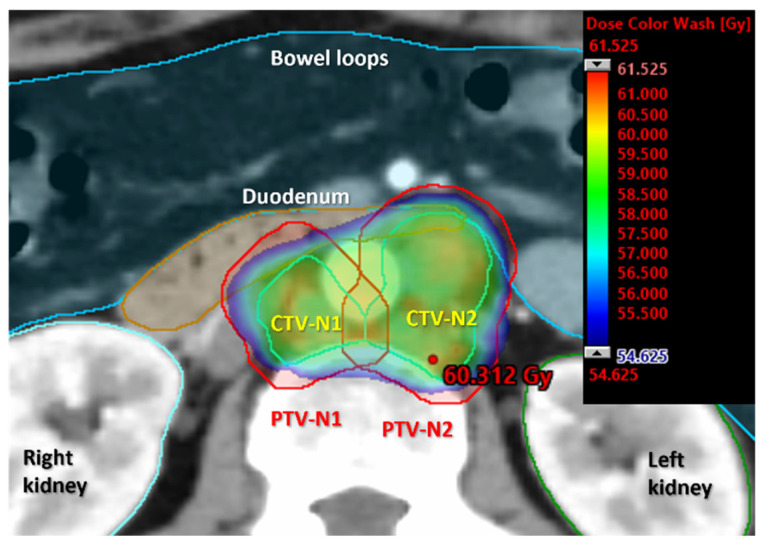
Coverage-probability-based treatment plan using VMAT for simultaneous integrated boost of two pathological para-aortic nodes (CTV-N1 and CTV-N2) located in close proximity to the duodenum. PTV-N is 5 mm margin from CTV-N. Boost dose of 57.5 Gy is prescribed, with the elective dose of 45 Gy in 25 fractions. Dose color wash represents 95–107% of the prescribed dose.

While nodal boosting has demonstrated remarkable benefits, concerns arise regarding increased radiation doses to OAR, such as the bowel and bladder, which may result in higher rates of toxicity and impaired quality of life (QoL). Significant nodal regression during treatment could also result in higher-than-planned radiation doses to OAR. It is particularly challenging, when applying boost to PAO nodes in close proximity to the duodenum, to deliver an appropriate tumoricidal dose to the target while limiting small bowel toxicity to acceptable levels. A single-institution retrospective study focusing on patients treated for gynecologic cancers with gross PAO metastases from 2005 to 2009 highlighted that most grade ≥2 duodenal toxicity arose in cases where duodenal V55 exceeded 15 cm^3^ and D2cc of 60 Gy [84]. Others have suggested dose thresholds of duodenal V55 Gy < 1 cm^3^ [85] or small bowel maximum doses of <55 Gy [86]. In recent years, the concept of coverage probability planning has been proposed and increasingly employed in clinical practice, which allows SIB delivery with a central dose peak and sharp dose fall-off at the perimeter of the nodal target volume, thereby reducing collateral high-dose volumes into the adjacent OAR [9]. Early clinical outcomes reported by Lindegaard et al. [16] demonstrated promising early clinical remission and a dose reduction of 5–6 Gy towards the surrounding OAR in both small and large nodes, independent of the anatomical position and intended dose level of SIB, ranging from 55–57.5 Gy in 25 fractions. Figure 1 and Figure 2 illustrate the dosimetric characteristics of coverage probability planning in VMAT SIB.

### 4.3. Simultaneous Integrated Boost in the Context of IGABT

The paradigm shift to IGABT has benchmarked improved pelvic control and survival, while the use of IMRT and image-guided radiotherapy (IGRT) has resulted in a reduction of toxicity. This has highlighted that the efficacy and toxicity of additional nodal boosting in definitive CRT must be interpreted in the context of contemporary standards of precision radiotherapy techniques and planning aims.

Key studies that have investigated SIB for dose escalation in the context of CRT with IGABT are summarized in Table 1, including a description of the study design, dose volume characteristics, and relevant clinical outcomes.

It is noteworthy that for many of these studies, the overall regional nodal control was reported instead of the tumoricidal effect of SIB on individual nodes. Moreover, the individual nodal characteristics, including dose volume parameters, maximum standardized uptake value (SUVmax), and location of failures with regards to boost targets, were not consistently reported. The definition of positive nodes on imaging was also variable, highlighting the potential for false positive or negative inclusion, which may influence the interpretation of clinical outcomes. The evaluation of prognostic factors for nodal control is often limited due to the low incidence of boost-target failures in most studies [8,10,11,16]. Despite the heterogeneity, among the studies that reported on SIB using contemporary techniques and nodal dose prescriptions mostly between 55–60 Gy in EQD2, excellent individual nodal control of above 90% was observed, with low incidence of severe toxicity [8,10,11,16,82,87].

Based upon favorable clinical outcomes demonstrated across studies, IMRT or VMAT to deliver dose escalation to pathological nodes detected on imaging, preferentially with SIB using coverage probability planning, is recommended by the ESGO/ESTRO/ESP guidelines [45]. Depending on nodal size and the expected dose contribution from brachytherapy, a total dose of approximately 60 Gy in EQD2 should be intended.

The topic of nodal dose escalation is being further investigated in the EMBRACE II study [88], for which nodal disease outcome is a primary endpoint. The study applies a standardized dose prescription of 55 Gy in 25 fractions to nodes within and 57.5 Gy to nodes outside the true pelvis using VMAT SIB, as well as coverage probability planning, and the results are highly anticipated. Meanwhile, mono-institutional experience following the EMBRACE II approach and planning objectives has reported encouraging outcomes of 2-year involved nodal control and regional nodal control of 99% and 93%, respectively, with a low incidence of severe treatment-related toxicity [8].

## 5. Elective Para-Aortic Radiotherapy

The concept of elective PAO irradiation is plausible, as patients with node-positive LACC often harbor microscopic subclinical disease in the PAO region, which may be sterilized by radiotherapy and spared from the potential morbidity and treatment delays associated with surgical lymph node staging. The RetroEMBRACE and EMBRACEI studies [6,7] have highlighted PAO failure as a significant challenge to regional control and cure, which account for approximately two-thirds of all nodal failures in patients with node-positive disease following CRT and IGABT, with the majority occurring outside of the elective target volume of pelvis EBRT. Elective PAO irradiation has demonstrated a positive effect on PAO recurrence-free survival [15,89,90,91], DFS [90], and even OS [15,89] from retrospective and prospective studies, as occult PAO metastasis may serve as sanctuary sites for the development of distant metastasis [89,90].

Early randomized trials conducted from the 1970s to the 1980s first examined the role of elective PAO radiotherapy. In the RTOG 79–20 study [92], 367 patients with stage IIB or bulky (≥4 cm) stage IB/IIA disease received standard pelvic radiotherapy with or without PAO radiotherapy. The ten-year overall survival was improved in the extended-field radiotherapy arm (55% vs. 44%, *p* = 0.02), but no difference in DFS was observed. For patients who had a complete response, there was a lower cumulative incidence of distant failure in the extended-field irradiation arm (*p* = 0.053) and a higher percentage of salvage in those who developed local failures. On the other hand, there was a higher incidence of grade 4 or 5 toxicities in the extended-field arm. It is noteworthy that the trial was conducted in the era before concurrent chemotherapy became the standard of care for LACC and the availability of modern radiotherapy techniques for OAR sparing.

More recent studies have also assessed the efficacy of elective PAO radiotherapy, despite significant heterogeneity in patient selection and results. Centers such as the Pittsburgh group [10] have systematically applied elective PAO IMRT 45 Gy in 1.8 Gy fractions for patients with PET-staged positive pelvic nodal disease and SIB 55 Gy to involved nodes. After a median follow-up of 29 months, the rate of PAO failure in patients with pelvic-only nodes was 2.5%, and the incidence of late grade ≥ 3 toxicity was 4%. Another two-center retrospective study in Taiwan [93] investigated the role of elective PAO radiotherapy for node-positive LACC without clinical PAO disease. Patients received elective PAO IMRT of 45–50.4 Gy in 1.8 Gy per fraction and SEB to involved pelvic nodes to 59.4 Gy. After a median follow-up of 63 months, PAO failure was effectively reduced from 23.7% in the pelvic radiotherapy group to 1.3% in the elective PAO radiotherapy group (*p* < 0.001). Compared to pelvic radiotherapy alone, elective PAO radiotherapy also significantly improved 5-year PAO recurrence-free survival (56.8% vs. 100%, *p* < 0.001) and cancer-specific survival (56.5% vs. 93.9%, *p* < 0.001) among patients with common iliac node or ≥3 pelvic nodes, but no survival difference was observed in patients who had pelvic lymph nodes below the common iliac bifurcation and 1–2 pelvic lymph nodes. There was no significant increase in severe toxicities. Other studies, on the other hand, failed to demonstrate a consistent clinical benefit. Analysis of 228 cervical cancer patients treated with CRT between 1999 and 2009 identified in two prospective databases in Canada showed no difference in the rate of PAO relapse, DFS, or OS with the addition of elective PAO radiotherapy 40 Gy to standard pelvic radiotherapy and concurrent cisplatin chemotherapy [94]. However, it is worth noting a lower dose of elective PAO radiotherapy was prescribed, as well as considerable ambiguity in the selection criteria, for which patients with enlarged pelvic lymph nodes, or equivocal pelvic nodes in the setting of a hypoxic primary tumor, were considered for elective PAO radiotherapy.

A recent meta-analysis [95] was performed to assess the role of elective PAO radiotherapy in cervical cancer in the context of CRT. Pooling patients from 3 cohorts consisting of 1113 patients, a modest but statistically significant association between PAO radiotherapy and DFS was found (pooled aHR 0.87, 95% CI: 0.79–0.97).

A drawback of adding PAO radiotherapy is the increased risk of toxicity, such as gastrointestinal effects and myelosuppression. While a high incidence of severe toxicity was reported in early RTOG trials [92,96,97] (14–49% grade ≥ 3 toxicity) that employed antiquated radiotherapy techniques, modern IMRT or VMAT has been associated with acceptable toxicity profiles (6–10% acute grade ≥ 3 gastro-intestinal (GI) toxicity and 6.5% late grade ≥ 3 GI toxicity) [98]. Notably, the overall volume receiving 43 Gy (V43Gy) strongly correlates with grade ≥ 2 GI morbidity [99]. With the employment of IMRT or VMAT techniques, attention to planning quality to achieve high dose conformity and the systematic application of daily image guidance for increased precision and smaller Planning Target Volumes (PTV), a significant reduction of V43Gy has been demonstrated to be feasible in clinical practice [77]. It is believed that elective PAO radiotherapy in patients with a high risk of occult nodal disease using modern techniques results in an acceptable toxicity profile.

With attempts to balance efficacy and potential toxicity, it is still a matter of debate whether elective PAO radiotherapy should be applied to all patients with node-positive LACC, irrespective of the involved nodal region or number. While consensus on selection criteria has not been achieved, commonly considered risk factors include the number of positive pelvic nodes and the presence of common iliac nodes [91,100,101]. Analysis of patients from the EMBRACE I study demonstrated that the presence of any common iliac node was an independent risk factor for PAO nodal failure, and that elective PAO radiotherapy was associated with a protective effect [91]. Apart from pelvic node involvement, risk factors such as the FIGO stage [102], the primary tumor size [91,100,102], the presence of pelvic side wall involvement [90], and elevated circulating squamous cell carcinoma antigen [102] have also been suggested.

Building upon these insights, the ongoing multicenter EMBRACE II study [88] has systematically integrated elective PAO irradiation in cases of node-positive LACC exhibiting high-risk features, such as three or more involved nodes or any involved node at or above the common iliac region. The upper boundary of the elective PAO region is set at the level of the renal veins, which is in keeping with studies showing that most PAO metastases occur below this level, and skip metastasis is exceedingly rare [101,103]. This study will provide prospective evidence on the value of elective PAO radiotherapy on overall disease control and toxicity in the context of contemporary radiotherapy techniques. Furthermore, a multicenter phase III randomized controlled trial (NCT03955367) [104] will investigate the role of elective PAO radiotherapy for patients with ≥2 pelvic lymph nodes, any pelvic lymph node with a short diameter of ≥1.5 cm, or parametrial involvement of the pelvic side wall, and assess clinical outcomes including the 3-year PFS, distant failure-free survival, OS, and toxicity profile.

Moving forward, patient selection for elective PAO irradiation may be further improved by nomograms [105,106] that predict PAO failure, and there is ongoing research exploring if the prediction accuracy could be improved by incorporating molecular tumor profiles or radiomic signatures.

## 6. The Evolving Landscape of Systemic Treatment and Leveraging Potential Synergy Between Radiotherapy and Immunotherapy

With excellent outcomes in local control and improved pelvic control achieved with MRI-guided adaptive brachytherapy, systemic failure has become a predominant challenge to cure for patients with node-positive LACC [6]. For the past 25 years, concurrent weekly cisplatin has been the standard CRT regime, despite efforts to improve outcomes with intensified systemic treatment, such as doublet chemotherapy, neoadjuvant or adjuvant chemotherapy, or targeted therapy combinations.

Earlier studies on the role of neoadjuvant chemotherapy before definitive radiotherapy were largely unsuccessful due to heterogeneity in intervention and inconsistent results [107]. A phase III study by Dueñas-González et al. [108] compared definitive radiotherapy with standard concurrent cisplatin to concurrent-adjuvant cisplatin-gemcitabine. The experimental arm showed a 9% improvement in 3-year DFS (74.4% vs. 65.0%, *p* = 0.029), as well as OS (HR 0.68, 95% CI 0.49–0.95; *p* = 0.0224, but a substantial 40% increase in the incidence of grade 3 or 4 toxicities (86.5% vs. 46.3%; *p* < 0.001); this study did not result in change in clinical practice. The phase 3 OUTBACK trial [109] revealed that four cycles of adjuvant carboplatin and paclitaxel chemotherapy following standard cisplatin CRT did not improve survival. The combinations of targeted therapy with standard CRT have yielded varied outcomes. While the addition of the anti-epidermal growth factor receptor (EGFR) monoclonal antibody cetuximab to CRT did not demonstrate improvement in clinical outcomes [110], the EGFR tyrosine kinase inhibitor (TKI) erlotinib [111] and anti-vascular endothelial growth factor (VEGF) inhibitor bevacizumab [112] have been shown to be safe when combined with radiation from respective phase II studies, but their benefits are yet to be established in randomized trials. The ribonucleotide reductase inhibitor, triapine, did not improve OS when combined with CRT in the phase III randomized trial NRG-GY006 [113].

On the other hand, the recently published INTERLACE trial [17], utilizing short-course weekly induction dose-dense carboplatin and paclitaxel for 6 weeks before standard cisplatin CRT, demonstrated statistically significant improvement in PFS (72% vs. 64%, HR 0·65 (95% CI 0.46–0.91); *p* = 0·013), and OS (80% vs. 72%, HR 0·60 (95% CI 0.40–0.91); *p* = 0.015) at 5 years compared to CRT alone. Grade ≥3 adverse events were 59% in the induction chemotherapy arm compared with 48% in the CRT alone arm. There is considerable debate as to whether the INTERLACE results should be routinely applied in the treatment of LACC [114]. It should be noted that the INTERLACE trial excluded patients with PAO disease or distal vaginal involvement, which are well-known high-risk factors of treatment failure. In addition, the INTERLACE trial was designed and initiated before the advances in image-guided adaptive radiotherapy (benchmarked by EMBRACE I), and around 60% of patients received 3D conformal EBRT and 70% had brachytherapy prescription to point A. Therefore, it is uncertain whether the INTERLACE regime was compared to a “less than optimal” control arm. In the INTERLACE trial, a tight window 7 days was applied from the completion of neoadjuvant chemotherapy to the initiation of CRT, to which the authors attributed limiting tumor repopulation as a potential reason for the positive outcomes achieved in this study, in contrast to historical studies on neoadjuvant chemotherapy. However, the increase in hematological toxicity in the neoadjuvant chemotherapy arm was associated with an 11% reduction in the proportion of patients who were able to complete the intended five cycles of concurrent cisplatin. It is unclear whether the expense of reduced concurrent cisplatin exposure could result in suboptimal outcomes in the real-world setting involving less fit patients and less flexible radiotherapy schedules.

The immunogenic potential of cervical cancer is underscored by the viral link to HPV [115], a high proportion of cases with the programmed cell death ligand 1 (PD-L1) overexpression [116], and a significant tumor mutational burden (TMB) [117], which reflects the abundance of neoantigens. Immune checkpoint inhibitors (ICIs) are the most abundantly studied class of immunotherapy agents in cervical cancer. ICIs such as pembrolizumab (anti-PD-1) [118], atezolizumab (anti-PD-L1) [119], and cadonilimab (bispecific antibody targeting PD-1 and CTLA-4) [120] have shown strong signals combined with platinum-based chemotherapy in the first-line treatment of metastatic or recurrent cervical cancer. Of note, pembrolizumab plus chemotherapy, with or without bevacizumab, has become the FDA-approved standard first-line therapy for patients with persistent, recurrent, or metastatic cervical cancer, whose tumors express PD-L1 based on the KN-826 study [121].

The intricate reciprocity between radiotherapy and immunotherapy has been elucidated in preclinical studies, which underscores the immunomodulatory effects of radiation by increasing the release of tumor neoantigens, facilitating T-cell tumor infiltration, and increasing the expression of immune checkpoint ligands, including PD-L1. With such a rationale, there has been strong enthusiasm in exploring radiotherapy-immunotherapy combinations. The phase 3 randomized double-blind trial CALLA [122] investigated the use of concurrent and adjuvant durvalumab with CRT administered for up to two years. In this study, there was no difference in PFS between the two treatment arms, but a trend towards PFS improvement in the subgroup of patients with FIGO-2009 stage ≥III disease with positive lymph nodes. On the other hand, the phase 3 randomized trial KN-A18 [18] demonstrated practice-changing results with the addition of pembrolizumab in the same concurrent-adjuvant fashion. Eligible patients were FIGO-2014 stage IB2–IIB with node-positive disease or stage III–IVA, regardless of nodal status; 89% of patients were treated with IMRT or VMAT radiotherapy techniques. Following a pre-specified second interim analysis and a median follow-up of 29.9 months, median OS was not reached in either group. The 36-month OS was 82.6% in the pembrolizumab–CRT group and 74.8% in the placebo–CRT group (HR 0.67, 95% CI 0.50–0.90; *p* = 0.004). More apparent OS improvement was detected in patients who had FIGO-2014 III-IVA disease (HR 0.57, 95% CI 0.39–0.83) compared to patients who had stage IB2-IIB disease with positive lymph nodes (HR 0.89, 95% CI 0.55–1.44). The toxicity profile was acceptable, with 78% and 70% of patients experiencing grade ≥3 adverse events in the pembrolizumab–CRT group and placebo-CRT groups, respectively, and mainly comprised of hematological events. Potentially immune-mediated adverse events occurred in 39% of patients in the pembrolizumab-CRT group and 17% in the placebo-CRT group. The results of this study led to FDA approval of pembrolizumab with CRT for patients with FIGO-2014 Stage III-IVA cervical cancer [123]. The discordant findings demonstrated by the KN-A18 and CALLA trials could be due to differences in patient composition, where a higher proportion of patients from KN-A18 had lymph node involvement (84% vs. 74%) or intrinsic differences in the mechanisms of drug action. Long-term outcomes from the KN-A18 trial are awaited.

While the KN-A18 trial demonstrated practice-changing results, there are still many unanswered questions, including the optimal immunotherapy agent, the ideal sequencing of immunotherapy and radiation, and the potential for clinical and molecular predictive biomarkers. Key studies investigating the combination of immunotherapy with definitive CRT in node-positive LACC are summarized in Table 2.

Two prospective trials involving atezolizumab [130] and cadonilimab [124] in the concurrent-adjuvant setting have completed accrual, and their results will soon elucidate whether a similar positive effect can be demonstrated with other immunotherapy agents beyond pembrolizumab. In addition, the phase II COLIBRI trial [129] evaluates the biological impact of neoadjuvant nivolumab plus ipilimumab before CRT, followed by maintenance nivolumab monotherapy. The interim results showed neoadjuvant dual immune checkpoint blockade was generally safe and resulted in significantly increased tumor-associated CD8+ cells, the CD8+/FOXP3+ ratio, and the ‘HOT’ score, suggesting potential in situ immune modulatory effects. The association between a high CD8+/FOXP3+ ratio and better clinical outcomes after neoadjuvant chemotherapy in cervical cancer [134], as well as the ‘HOT’ score with immunologically active tumors [135], have been described previously. The clinical endpoints of PFS and OS will be reported in 2025. On a separate note, other forms of immunotherapy, such as therapeutic vaccines targeting HPV oncogenes, are also being explored. In the single-arm, phase II IMMUNOCERV trial [127], a subcutaneously administered HPV-specific vaccine (PDS0101) containing peptide pools encoding E6 and E7 antigens was administered to patients who received definitive CRT for LACC. Although the trial was terminated early due to a change in the standard of care, intriguing early-tumor responses during and metabolic remission after CRT were observed. Targeted immunotherapy approaches, such as HPV-directed vaccines, may be a promising direction in the management of node-positive LACC.

## 7. Future Directions in Personalized Therapy

### 7.1. Maximizing the Therapeutic Ratio of Radiotherapy

While treatment intensification strategies have led to improvement in disease control at local, regional, and distant sites [6,7,17], they are often associated with an increase in treatment-related toxicity. Precision radiotherapy techniques, such as IGRT, VMAT, or IMRT planning, have allowed a reduction in treatment volumes and toxicity [77]. While high-grade toxicity is uncommon in contemporary radiotherapy techniques, low-grade morbidity, some persistent, often occurs following definitive CRT and may have an adverse impact on QoL in patients with prospects for long-term survival [98,136,137]. It is important to better characterize dose-volume effects of radiotherapy, including bowel, urinary, and, although often underreported, bone marrow domains, from both physician- and patient-reported outcomes and QoL assessments. The ongoing EMBRACE II study [88] will prospectively evaluate these aspects and further elucidate strategies to enhance the therapeutic ratio by maximizing tumor control and minimizing toxicity.

External beam radiotherapy for LACC is susceptible to uncertainties from both inter-fractional and intra-fractional anatomical changes from tumor response and internal organ motion. These uncertainties contribute to the PTV margins required, which in turn influence the PTV volume, OAR dose, and treatment morbidity. In the EMBRACE I study [69], where a broad variation in EBRT practices was observed across institutions, the utilization of IMRT and image guidance was limited, and PTV margins of 8–10 mm were commonly applied for setup uncertainties, with patient positioning based on skin marks. In contrast, the EMBRACE II study [88] systematically applies the IMRT/VMAT technique, with daily IGRT, reduced 5-mm PTV margins, and high dose conformality. Analysis of the initial 132 patients in the EMBRACE II study [77] demonstrated that, compared to EMBRACE I, there was a reduction in median V43Gy for pelvic irradiation by 972 mL (41%) and 1130 mL (39%) for patients receiving pelvic and para-aortic (PAO) irradiation. Mitigating V43Gy during EBRT is clinically relevant, as it has been shown to be associated with both acute and late bowel morbidity [138,139]. Another dosimetric study [140] demonstrated that further reducing the PTV margin from 5 to 0 mm and daily online image guidance resulted in a decrease of bowel V30 Gy by 13%, body V43 Gy by 19%, and PTV by 39%, while maintaining robust elective lymph node target coverage against translational and rotational setup errors. Moreover, adaptive radiotherapy strategies, such as Plan-of-the-Day (PotD) and fully online-adaptive approaches involving daily segmentation and re-optimization, have been explored. A single-institution study [141] showed PotD enabled a reduction in PTV volume by a median of 250 cm^3^ compared to no adaptation, while fully online-adaptive methods could further decrease the PTV volume by 144 cm^3^. While considerable reductions in irradiated volumes have been made possible by IGRT techniques, further research is necessary to evaluate their clinical significance and define the optimal strategy.

Although strategies such as adaptive radiotherapy with standard IMRT or VMAT have been proposed, they have not been widely adopted due to the limited resolution of cone beam CT imaging and impracticalities concerning frequent replanning. MR-guided radiotherapy (MRgRT) using MR Linac enables more accurate visualization of gynecologic tumors and OAR and has been shown to be advantageous in terms of tailoring target volumes to inter-fractional anatomical changes and monitoring intra-fractional motion [142,143]. This improved soft tissue definition and treatment precision could enable further reductions in target volumes and normal tissue sparing, both in the definitive and salvage settings, such as stereotactic ablative radiotherapy for limited abdominopelvic nodal recurrence [144]. On the other hand, drawbacks in the utilization of MR Linac in LACC include field-size limitations that could be frequently encountered in PAO radiotherapy, leading to additional technical complexities involved in treatment planning and verification [145]. Further research is necessary to evaluate the potential of MR Linac-based EBRT in cervical cancer and whether the technical advantages could translate into improved clinical outcomes. Furthermore, autosegmentation and deep learning algorithms may offer effective solutions to navigate the complexities and improve efficiency in adaptive radiotherapy.

Proton therapy (PT) is an emerging radiotherapy modality that allows for highly localized dose deposition due to its unique physical property. Intensity Modulated Proton Therapy (IMPT) has shown dosimetric advantages in LACC, with significant reductions in intermediate and low dose spillage, including pelvic bone Dmean and bowel bag V15Gy compared to IMRT or VMAT [146]. Single institutional experiences have also reported on the opportunity of applying IMPT in settings such as extended-field PAO radiotherapy [147] and pelvic re-irradiation [148], with promising toxicity outcomes. The highly anticipated prospective PROTECT trial [149], being conducted in The Netherlands, aims to compare the effects of IMPT with IMRT or VMAT photon therapy on dose-volume parameters and treatment-related morbidity for LACC patients undergoing CRT. This study would be the first to reveal the potential of IMPT in clinical practice to reduce the OAR dose and improve the toxicity profile and QoL for patients with LACC. Furthermore, with the potential advantage of IMPT in terms of bone marrow sparing, the effect of IMPT and IMRT or VMAT on the local and systemic immune system is evaluated, as measured by the bone marrow activity on MRI scans using the Dixon technique and the number and function of circulating leukocytes (myeloid cells and lymphocytes). This study will shed light on the clinical value of IMPT in the modern era of precision radiotherapy.

### 7.2. The Potential for Predictive Biomarkers for Personalized Treatment

The future of oncology development is leaning toward personalized treatment approaches tailored to individual risk profiles. A crucial challenge to this approach is the limitation of traditional clinicopathological risk factors in the reliable identification of patients at high risk of different failure types. These prognostic factors (such as stage, lymph node status, and histology) identified to date have poor ability to predict relapse [150]. Analysis from the RetroEMBRACE trial [6] showed that 11.3% of the relatively low-risk patients (FIGO I-IIB, node-negative) experienced systemic failure, while a significant proportion (63.6%) of patients with isolated PAO relapse had node-negative disease at diagnosis. In the last decade, there has been growing interest in the integration of radiomic signatures or biological biomarkers to traditional clinicopathological factors to more accurately define risk groups and assess the anticipated response to treatment, hence the potential for more precise risk-adapted treatment approaches, such as intensification or de-intensification.

Radiomics has been demonstrated to be a promising non-invasive method by which to identify nodal metastasis in LACC that is below the sensitivity threshold of standard imaging [151]. Examples include radiomic models developed from PET-CT [152] or MRI [153] data integrated with clinical parameters to improve the diagnostic sensitivity of pathological lymph nodes compared to traditional metabolic or morphological features. Moreover, there is also potential in radiomic signatures to predict treatment responses [154,155,156] and survival outcomes [157]. This may add to the predictive accuracy of clinical nomograms in selecting patients for PAO irradiation [158]. However, the field remains in its infancy, with ongoing challenges related to reproducibility and model robustness, necessitating further research to validate these innovative approaches.

Molecular and serum biomarkers have also demonstrated potential in disease prognostication or response prediction. One multicenter randomized phase II study found that tumors with high tumor expression of the hypoxic marker CA9 had higher risk of PAN relapse after CRT, and although extended-field radiotherapy improved PAO recurrence-free survival overall, the therapeutic efficacy of extended-field radiotherapy in terms of improved DFS was only observed in CA9-only positive LACC, but not in CA9/HIFs double-positive patients [159,160]. From the BIOEMBRACE study [161], p16 negative status and tumor necrosis on MRI were independently associated with a poor response to CRT, whereas PD-L1 expression and L1CAM ≥ 50% had an independent adverse impact on local and pelvic control. Other studies have reported the pre-treatment platelet-to-lymphocyte ratio [162] to be associated with poorer survival, and higher pre-treatment serum squamous cell carcinoma antigen levels [163] have been associated with higher risk of distant recurrence after CRT.

The role of plasma HPV-circulating tumor DNA in predicting outcomes following CRT has been studied, with compelling evidence reported in a prospective multicenter validation study in Canada [164]. Patients with detectable HPV circulating tumor DNA on digital polymerase chain reaction or next-generation sequencing at the end of, 4–6 weeks after, and 3 months after CRT had significantly worse 2-year PFS compared with patients with undetectable levels. It is well known that effective salvage therapy is not possible for the majority of cervical cancer patients who experience treatment relapse [165]. Hence, HPV circulating tumor DNA testing is a promising biomarker for the early identification of patients with molecular residual disease at the end of CRT, who are at high risk of clinical recurrence and may be candidates for treatment intensification. Further studies are eagerly awaited to validate this biomarker-selected, risk-adapted approach.

## 8. Conclusions

Node-positive cervix cancer is a challenging entity characterized by a high risk of relapse after definitive CRT, and a high unmet need exists to optimize outcomes. With a paradigm shift towards IGABT to achieve excellent local and pelvic control, regional nodal and distant failure represent the predominant failure pattern. Various treatment intensification strategies, such as lymph node surgery and radiotherapy dose escalation beyond standard CRT, have been explored. Evolution in EBRT techniques has enabled the precise delivery of a simultaneous integrated boost to pathological nodes and elective PAO radiotherapy to subclinical disease, with improvement in regional control without significant additional toxicity. Novel life-prolonging immunotherapy-CRT combinations have transformed the landscape of systemic therapy. While ongoing research to further enhance the therapeutic ratio and define the optimal combination of radiation with systemic therapy is anticipated, and there is great promise for the development of predictive biomarkers that allow personalized, risk-adapted treatment approaches.

## Figures and Tables

**Table 1:** Key studies investigating simultaneous integrated nodal boost in the context of CRT with IGABT.

Study (Design)	N of Patients (% PAO+)	N of Boosted Nodes	Median N of Nodes per Patient (Range)	%PET Staged	RT Technique	Elective PAO Irradiation (Criteria)	Median Boost Dose †	INC	RC/LRC/DFS	Grade 3+ Toxicity	Poor Prognostic Factors on RC
Vargo et al. 2014 (Retrospective) [10]	61 (33% PAO+)	179	2 (1–16)	100%	IMRT	Yes (Pelvic node +)	55 Gy *EQD2 55.9 Gy*	99% at 29 months	RC at 3 years: 94% DFS at 3 years: 57%	Late overall: 4%	None identified
Lindegaard et al. 2017 (Retrospective) [16]	23 (%PAO+ not specified)	74	2 (1–10)	100%	VMAT	Yes (>2 pelvic node + OR common iliac node +)	Small pelvis: 55 Gy *EQD2 55.9 Gy* Common iliac/PAO: 57.5 Gy *EQD2 58.9 Gy* One large node: 60 Gy *EQD2 62 Gy*	99% at 3 months	RC at 9 months: 87%	Not examined	Not analyzed
Dang et al. 2019 (Retrospective) [11]	74 (46% PAO+)	Not specified	3 (1–7)	0%	IMRT	No	62.5 Gy *EQD2 65.1*	100% at 3 years	Not examined	Acute GI/GU: 8.1%/0% Late GI/GU: 5.5%/0% One G4 rectovaginal fistula	Not analyzed
Jethwa et al. 2020 (Retrospective) [12]	59 (44% PAO+)	Not specified	3 (2–6)	93%	VMAT	Yes (Common iliac node +)	56.25 Gy *EQD2 57.4 Gy*	95% at 30 months	LRC at 3 years: 89%	Acute GI/GU: 3%/0% Late GI/GU: 6%/ 6%	Not analyzed
Jayatilakebanda et al. 2021 (Retrospective) [13]	23 (22% PAO+)	Not specified	2 (range not specified)	70%	IMRT /VMAT	Yes (Common iliac node +)	60 Gy *EQD2 62 Gy*	Not specified	RC at 3 years: 79%	Overall: 0%	Not analyzed
Tiwari et al. 2021 (Prospective comparative—boost versus no-boost) [14]	71 in boost arm (18% PAO+)	Not specified	<3 pelvic nodes (range not specified)	<7%	IMRT /VMAT (40%) 3D-CRT (60%) SIB (40%) SEB (60%)	No	55–57.5 Gy (SIB) *EQD2 55.9*–*58.9 Gy*	Not specified	RC at 3 years: 93%	Acute GI and GU: 7% Late GI and GU: 2.8%	Pelvic nodal volume ≥ 3 cc Nodal diameter ≥ 2 cm Boost dose ≤ 58 Gy
Lee et al. 2022 (Retrospective) [82]	115 (%PAO+ not specified)	411	2 (1–15)	100%	3D-CRT (66%) IMRT (27%) Combined (7%) SIB (27%) SEB (66%) SIB+SEB (7%)	No	*EQD2 53.1 Gy*	94% at 2 years	RC at 44 months: 85.2%	Overall: 12% One grade 4 duodenum ulcer perforation	Neutrophil-to-lymphocyte ratio ≥3.1 Total radiation dose Initial nodal volume ≥5.29 mL
Cheung et al. 2023 (Retrospective) [8]	54 (39% PAO+)	234	3 (1–16)	76%	VMAT	Yes (>2 pelvic node + OR common iliac node +)	Small pelvis: 55 Gy *EQD2 55.9 Gy* Common iliac/PAO: 57.5 Gy *EQD2 58.9 Gy*	99% at 2 years	RC at 2 years: 93% DFS at 2 years: 78%	Acute GI/GU: 2%/0% Late GI/GU: 2%/2%	Adenocarcinoma histology Primary tumor SUVmax (on LRC)

† Equivalent dose in 2 Gy per fraction (EQD2), assuming an α/β ratio of 10 Gy for tumor, not accounting for dose contribution from brachytherapy. Abbreviation: N = number; PAO = para-aortic; PET = positron emission tomography; RC = regional control; INC = individual nodal control; LRC = locoregional control; DFS = disease-free survival; IMRT = intensity-modulated radiation therapy; VMAT = volumetric modulated arc therapy; 3D-CRT = 3-dimensional conformal radiation therapy; SEB = sequential boost; SIB = simultaneous integrated boost; GI = gastro-intestinal; GU = genito-urinary; SUVmax = maximum standardized uptake value.

**Table 2:** Key studies investigating the combination of immunotherapy agents with definitive CRT involving node-positive LACC.

Setting	Study Status	Trial Identifier	Study Location	Phase	Immunotherapy Class	Experimental Arm	Control Arm	Primary Outcomes/Results (If Available)
Conc./Adj.	Completed	CALLA (NCT03830866) [122]	International	III	Anti-PD-L1	Durvalumab + CCRT	Placebo + CCRT	No PFS improvement
Conc./Adj.	Active, not recruiting	KEYNOTE-A18 (NCT04221945) [18]	International	III	Anti-PD-1	Pembrolizumab + CCRT	Placebo + CCRT	Median OS not reached in either arm after mFU of 29.9 months. 3-year OS 82.6% (95% CI 78.4–86.1) and 74.8% (95% CI 70.1–78.8) in the experimental and control arms, respectively. HR 0.67 (95% CI 0.50–0.90; *p* = 0.0040) 2-year PFS 67.8% (95% CI 62–73) and 57.3% (95% CI 51–63) in the experimental and control arms, respectively. HR, 0.70; 95% CI, 0.55–0.89; *p* = 0.0020
Conc./Adj.	Active, not recruiting	AK104-305 (NCT05235516) [124]	China	III	PD-1/CTLA-4 bispecific antibody	Cadonilimab + CCRT	Placebo + CCRT	PFS
Conc./Adj.	Active, not recruiting	ATEZOLACC (NCT03612791) [125]	France	II	Anti-PD-L1	Atezolizumab + CCRT	CCRT	PFS
Conc./Adj.	Recruiting	(NCT05084677) [126]	China	II Single-arm	Anti-PD-1	Toripalimab + CCRT → consolidation chemo and Toripalimab	N/A	ORR
Conc./Adj.	Prematurely closed	IMMUNOCERV (NCT04580771) [127]	US	II Single-arm	Immune nanoparticle liposomal HPV-16 E6/E7 multipeptide vaccine	PDS0101 + CCRT	N/A	Acute grade 3+ AE: 47%
Conc./Adj.	Completed	NiCOL (NCT03298893) [128]	France	I	Anti-PD-1	Nivolumab + CCRT	N/A	3 (19%) patients with grade 3 dose-limiting toxicities.
Neoadj./Adj.	Active, not recruiting	COLIBRI (NCT04256213) [129]	France	II Single-arm	Anti-PD-1 Anti-CTLA4	Nivolumab + Ipilimumab → CCRT → Nivolumab	N/A	Neoadjuvant nivolumab + ipilimumab is safe and potentially promotes de-novo immune-modulating activity
Neoadj./conc. OR conc.	Active, not recruiting	NRG-GY017 (NCT03738228) [130]	US	I	Anti-PD-L1	Atezolizumab → Atezolizumab + CCRT	Atezolizumab + CCRT	Immune Response
Adj.	Recruiting	eVOLVECervical (NCT06079671) [131]	International	III	PD-1/CTLA-4 bispecific antibody	CCRT → adj. Volrustomig	Placebo	PFS
Adj.	Active, not recruiting	ATOMICC (NCT03833479) [132]	Spain and Turkey	II	Anti-PD-1	CCRT → Dostarlimab	No further treatment	PFS
Adj.	Completed	GOG-9929 (NCT01711515) [133]	US	I	Anti-CTLA4	CCRT → Ipilimumab	N/A	Ipilimumab following CRT resulted in anti-tumor immune response

Abbreviations: Neoadj. = neoadjuvant; Conc. = concurrent; Adj. = adjuvant; PD-1 = programmed cell death protein 1; PD-L1 = programmed death-ligand 1; CTLA-4 = cytotoxic T-lymphocyte-associated protein 4; HPV = human papillomavirus; CCRT = concurrent chemoradiotherapy; N/A = not applicable; PFS = progression-free survival; ORR = objective response rate; mFU = median follow-up.

## Data Availability

Not applicable.
